# Multimorbidity research challenges: where to go from here?

**DOI:** 10.15256/joc.2011.1.9

**Published:** 2011-12-27

**Authors:** Gillian E. Caughey, Elizabeth E. Roughead

**Affiliations:** ^1^Quality Use of Medicines and Pharmacy Research Centre (QUMPRC), Sansom Institute, School of Pharmacy and Medical Sciences, University of South Australia, Adelaide, SA, Australia

**Keywords:** chronic conditions, comorbid, comorbidity, multimorbidity, multiple morbidity

The last decade has seen an increase in research focusing on patients with multiple chronic conditions. A recent review reported only two published studies on multimorbidity between 1990 and 2000, increasing to 39 between 2001 and 2010 [1]. This research has been largely driven by two factors: the increasing demographics of the older population worldwide [2], coupled with a growing awareness of the high prevalence of patients with multiple chronic conditions [1, 3]. Through this research, there has been an increasing appreciation and understanding of the complexities associated with the management and care of these patients both from a health practitioner and a patient perspective [4, 5]. However, there is still much work to be done if we are to improve health outcomes, quality of care, and develop the necessary healthcare systems required to provide integrated and coordinated care for this growing population. In order to move this research agenda forward, it is important to reflect on what we currently know, to facilitate the identification of potential gaps in our knowledge base.

The research to date has been very successful identifying the epidemiology of comorbidity and multimorbidity, and the problems and consequences of multimorbidity. For the older population, in particular, the presence of multiple conditions is common. The estimated prevalence of those aged 65 years and older with two or more chronic conditions is from 55% to over 80% [1, 6]. Over half of these patients will have a comorbid condition that will result in a treatment conflict with the potential to cause harm, complicating management [7–9]. Over half of patients with comorbidity simply do not ‘fit’ into current clinical guidelines. Multimorbidity is associated with decreased quality of life, self-rated health, mobility, and functional ability. It is also associated with increases in hospitalizations, physiological distress, use of healthcare resources, mortality, and costs [1, 6].

A number of factors contributing to the problems associated with multimorbidity are now also beginning to be understood. This includes a lack of evidence-based information to guide clinicians when caring for patients with multiple conditions. Most clinical guidelines fail to address the treatment of comorbid conditions [10]. Only 12% and 44% of clinical guidelines examined in Australia and the USA, respectively, made specific recommendations for patients with multiple comorbid conditions [10, 11]. The use of disease-specific guidelines for this population may also be associated with detrimental effects, difficult, complicated, and sometimes inappropriate treatment regimens [10]. Primary care clinicians struggle with the uncertainty of applying disease-specific guidelines to their patients with comorbidity [12] whilst patients struggle to adhere to complex treatment regimens [12]. Moreover, the evidence on which most guidelines are developed is from relatively short-term randomized clinical trials of single conditions where the elderly or those with comorbidity are excluded. Presently, the data required for the inclusion of evidence-based treatment of patients with multiple conditions into the development of clinical guidelines are limited or absent and urgently need addressing. It is essential that future studies generate this evidence base in a ‘real-world’ setting, where comorbidity and the older patient are included.

Coordination of care has also been identified as one of the major challenges for managing patients with multiple conditions [13]. Patients will often receive fragmented, inefficient, and ineffective care [14]. Current healthcare systems are hard to navigate, not only for patients but also health professionals. There is also little continuity or integration of care across primary, secondary, tertiary, and community healthcare sectors [13], yet models that specifically overcome this are still not clear. Patients will see a spectrum of health professionals ranging from their primary care physician to those in the acute care (hospital) setting, and inter-professional communication is suboptimal [14].

A clash of preferences and priorities, between patients and healthcare providers, and between healthcare providers, is also a contributing factor to the problems associated with multimorbidity [13]. A recent study showed that on almost 30% of occasions, the patient’s main priority was not in the top three priorities of their physician. This discordance was greater when patients had multiple conditions or had more competing demands [15]. There is also much inter-individual variability as to what conditions or health outcomes are of greatest importance when competing priorities are present [16]. How to best deal with these conflicting priorities is yet to be established.

Solutions to some of these problems are being trialled. Models of practice for patients with multimorbidity to deliver collaborative patient-centred care, which is coordinated and integrated, have been developed [17, 18]. These include a nominated care coordinator from within the healthcare team, a well-communicated care plan that includes patient circumstance, preferences, and goals. Importantly, these have been shown to improve both quality of life and care [18]. The effects on long-term health outcomes and healthcare costs and their implementation and dissemination into healthcare systems are still to be determined.

One of the key components to these models of care includes the consideration of patient circumstance and preferences into treatment and care plans. However, there are currently no agreed approaches to enable and support joint decision-making. This is particularly important for patients with multiple conditions with competing priorities, where the treatment of one condition may adversely affect another condition. Incorporating competing priorities into the decision-making process requires identification of benefits and harms under different treatment options across all conditions present. Presenting this information in terms of global health outcomes for patients, such as symptom relief, mobility, or survival, rather than disease-specific outcomes, is required so patients can achieve their most desired outcome [19]. How to best elicit and incorporate competing priorities and patient preferences into agreed care plans between health practitioners and patients has not been explicitly investigated. This research is essential if we are to provide reliable and reproducible guidance for the translation into patient-centred models of practice for multimorbidity.

The challenge now for the research enterprise is to further the understanding and develop solutions within a comprehensive framework that best addresses the needs of those with multimorbidity. Adapting the Australian framework for improving use of medicines may serve as a starting point. This multifaceted framework highlights the need for policy development, provision of objective information, education and training, service delivery, and interventions, as well as health systems monitoring, coordination, and facilitation [20]. Research addressing the needs of each of these areas, across multiple levels of health sector organization, primary, secondary, tertiary, and public health, and addressing the clinical, social, economic, and environmental dimensions will be necessary. The challenge is large but the scope of the problem is now clear, and with the increasing burden of multimorbidity to both individuals and society moving quickly toward us, this work will be critical to the future health of all.
